# Education of the "Rank and File."

**Published:** 1906-07

**Authors:** J. P. Corley

**Affiliations:** Greensboro.


### EDUCATION OF THE "RANK AND FILE."

By J. P. Corley, D. D. S., Greensboro.

(Alabama Dental Association.)

In science, politics, and religion the status is set and the present good to humanity determined—not by the tireless genius who stands in the vanguard of progress, but rather by the unknighted and unknown toiler in the "rank and file."

A duty, then, of the leader in thought is to make every legitimate effort to introduce into common practice the improvements which his more fortunate environments and personal industry and ambition have made his own. It has gotten to be a common custom to speak of the wonderful progress which has been made in dental science, and every intelligent man is proud of the advance which the profession has made; but is his high standard maintained by the "lay dentist?"

I am familiar with the methods practiced by many of the men of my own State, and, in fact, throughout the South, seeing, as I do, patients from almost all sections of the country, and I say with a blush that too many of them cut away the labial to reach small approximal cavities; extract teeth that could be made useful for a lifetime; adjust crowns with ill-fitting bands, and—horror of horrors!—put gold crowns on front teeth; crown teeth which would be better for an inlay or a filling; leave overhanging amalgam; introduce unserviceable and unhygienic bridge work; brush off stains and soft accretions which the patient could, and should, do every day; do patchwork for a patient year after year without instructing him in the care of his teeth, much less requiring a moderately sanitary condition of the mouth; and many other things which would have put to shame our professional grandfathers. Dental manufacturers still make forceps by the thousand and sell teeth by the one hundred sets.

Why, I ask myself and you, does the average practitioner thus trample upon his birthright? The reasons are many, but I shall enumerate only a few. The man usually knows better, but his patient does not. He, therefore, does not demand it—nay, he often does not even desire it—and the dentist, being a coward and a hireling, sells his services as a merchant sells goods—nay, worse, for the honest merchant sells his customer only that which is best suited to his needs. He sells himself—a slave to do his master's bidding. Small wonder that we get scant professional recognition! An erroneous conception of ethics often makes us condone, or at least fail to censure, that which we know to be gross malpractice—such as the extraction of teeth for the purpose of regulating and other reasons, gold crowns on front teeth, not requiring oral sanitation, etc.—so that our patients and their posterity might guard against such in the future.

We are too prone to an unchristian jealousy which makes us keep our knowledge to ourselves instead of putting forth extra effort to influence our unfortunate brother to cultivate a professional spirit.

While the man who is a member of the State Association is, as a rule, better than the man who is not, still the best that can be said of some of our members is that they are but carpenters. Recently an old member made the boast that in a previous month he had extracted eighty-five dollars' worth of teeth! You will not be suprised when I tell you that the price for extraction in that town is fifty cents per tooth, twenty-five cents in "job lots," and that a 24x36 inch "odontunder" sign hangs in front of his door and bears this sign: "Without pain!"

However, it is safe to say that a majority of the better element are members of the State Association, and that it is, and will continue to be, a wholesome influence in the education and professionalization of the "rank and file."

